# Modification of V_2_O_5_-WO_3_/TiO_2_ Catalyst by Loading of MnO*_x_* for Enhanced Low-Temperature NH_3_-SCR Performance

**DOI:** 10.3390/nano10101900

**Published:** 2020-09-23

**Authors:** Xianlong Zhang, Qinchao Diao, Xiaorui Hu, Xueping Wu, Kesong Xiao, Junwei Wang

**Affiliations:** 1 School of Chemistry and Chemical Engineering, Hefei University of Technology, Hefei 230009, China; Zhangxianlong@hfut.edu.cn (X.Z.); 15156218806@163.com (Q.D.); huxiaorui0917@163.com (X.H.); 2 Anhui Province Key Laboratory of Advanced Catalytic Materials and Reaction Engineering, Hefei University of Technology, Hefei 230009, China; 3 Instrumental Analysis Center, Hefei University of Technology, Hefei 230009, China; xiaokesong@126.com; 4 College of Chemistry and Chemical Engineering, Anqing Normal University, Anqing 246011, China

**Keywords:** NH_3_-SCR, V-W/Ti, low-temperature, manganese oxides, in-situ growth

## Abstract

V_2_O_5_-WO_3_/TiO_2_ as a commercial selective catalytic reduction (SCR) catalyst usually used at middle-high temperatures was modified by loading of MnO*_x_* for the purpose of enhancing its performance at lower temperatures. Manganese oxides were loaded onto V-W/Ti monolith by the methods of impregnation (I), precipitation (P), and in-situ growth (S), respectively. SCR activity of each modified catalyst was investigated at temperatures in the range of 100–340 °C. Catalysts were characterized by specific surface area and pore size determination (BET), X-ray diffraction (XRD), temperature programmed reduction (TPR), etc. Results show that the loading of MnO*_x_* remarkably enhanced the SCR activity at a temperature lower than 280 °C. The catalyst prepared by the in-situ growth method was found to be most active for SCR.

## 1. Introduction

Nitrogen oxides (NO*_x_*) are significant atmospheric pollutants released from industrial facilities leading to a serious impact on both air quality and human health, such as acid rain, photo-chemical smog, and ozone hole [1,2]. Among the technologies for NO*_x_* abatement, selective catalytic reduction (SCR) with NH_3_ is currently the most promising technology for eliminating NO*_x_* owing to its efficiency, economy, and selectivity [3,4]. Commercial V_2_O_5_-WO_3_/TiO_2_ has been extensively used as an industrial catalyst possessing stable catalytic performance in the temperature range of 300–400 °C with resistance to sulfur oxide poisoning. However, there are still several defects, such as low activity and poor N_2_ selectivity at lower temperatures restricting its more extensive applications [5,6]. Therefore, the study of new SCR catalysts available for wide temperature windows especially with high activity at low temperature has attracted extensive attention.

Low-temperature (150–250 °C) SCR process is preferable because it avoids pre-heating of flue gas in many cases and matches easily with the existing burner systems [7]. Owing to their particular features, various transition metals (Cr, Mn, Fe, Cu, and Ce) have been studied as the active components for SCR catalysts [8,9]. It was widely reported that manganese oxides showed outstanding SCR activity at lower temperatures. It was suggested that various types of labile oxygen species and diversiform oxidation states of manganese (Mn_3_O_4_, Mn_2_O_3_, MnO_2_, MnO) enable a multiple reaction routine of SCR at lower temperatures [10,11]. However, Mn-based catalysts are easily poisoned and subsequently inactivated in the environment of SO_2_ and H_2_O. It seems to be an interesting work to combine the low-temperature SCR activity of MnO*_x_* and stable sulfur resistance of V_2_O_5_-based catalysts to obtain a new catalyst with reliable performance in a wider temperature range.

Liu and co-workers [12] studied the effect of Mn on V_2_O_5_/TiO_2_ for NH_3_-SCR. They found that adding Mn significantly enhanced the activity, redox cycle, and the reactive intermediates, thus promoting the NH_3_-SCR performance. Zhao et al. [13] used transition metals (Cu, Fe, Mn, Co) to modify V_2_O_5_-based catalysts for the SCR of NO with NH_3_. The introduced metals induced the formation of vanadates, which helped disperse the V species.

In many cases, the SCR performance of catalyst primarily depends on the preparation methods due to conceivable differences in formation and dispersion states of surface-active species [14,15]. Therefore, it is meaningful to discuss the effects of the dispersion degree of accompanied surface-active species on the low-temperature SCR. In the past years, various methods, including impregnation, precipitation, sol-gel method, etc. have been widely used to prepare Mn-based SCR catalysts [16]. However, for traditional catalyst preparation methods (such as impregnation, precipitation, and sol-gel), thermal calcination is an essential step to obtain MnO*_x_* specie from decomposition of precursors. Agglomeration of surface particles caused by sintering often leads to poor dispersion of surface MnO*_x_* species especially at high loading contents resulting in a low catalytic efficiency [17,18]. In previous work, we have introduced an in-situ growth method with an efficient and facile strategy avoiding thermal calcination to controllably prepare supported MnO*_x_* catalysts.

Therefore, the impregnation method, precipitation method and in-situ growth method were used to modify the low-temperature SCR performance of V_2_O_5_-WO_3_/TiO_2_ catalyst by loading of MnO*_x_*. The chemi-physical properties of the obtained catalyst and SCR performance were measured for evaluating the influence of the preparation method on catalyst’s performance, as well for the exploration into the structure–activity relationship (SAP).

## 2. Materials and Methods

### 2.1. Materials

The V_2_O_5_-WO_3_/TiO_2_ catalysts were purchased from Hubei Honghu Siboying Environmental Protection Technology co., LTD (China). The main chemical composition of this catalyst was 0.8 wt.% V_2_O_5_, 2.02 wt.% WO_3_, 93.46 wt.% TiO_2_, 1.18 wt.% SiO_2_. The catalysts were crashed and sieved to particles with a size of 0.425–0.850 mm for further preparation steps.

### 2.2. Catalyst Preparation

#### 2.2.1. Precipitation Method (C)

V_2_O_5_-WO_3_/TiO_2_ catalyst was firstly immersed in Mn(NO_3_)_2_ solution with a certain concentration for 24 h. Then, the K_2_CO_3_ solution was gradually added with thorough stirring. The mixtures were placed at room temperature for 12 h and then filtered, followed by washing with de-ionized water 8–10 times. The resulting solid products were dried at 50 °C for 3 h and 110 °C for 12 h, and calcined in air flow at 400 °C for 3 h to obtain the modified catalysts noted as Mn-V-W/Ti-P.

#### 2.2.2. Incipient-Wetness. Impregnation (I)

The catalyst of V_2_O_5_-WO_3_/TiO_2_ was firstly impregnated with Mn(NO_3_)_2_ solution with certain concentrations by the pore-volume impregnation method. The wet samples were dried at 50 °C for 3 h and dried at 110 °C for 12 h. Finally, the particles were calcined in a vertical furnace at 400 °C in air flow for 3 h. The obtained catalyst was noted as Mn-V-W/Ti-I.

#### 2.2.3. In-Situ Growth Method (S)

**Figure 1:**
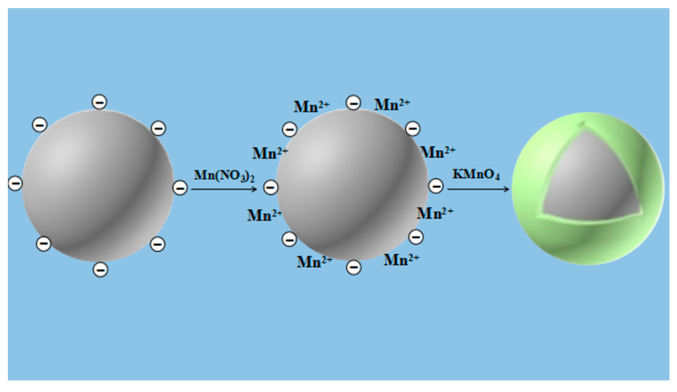
Schematic illustration of the synthesis process of the in-situ growth method.

Firstly, V_2_O_5_-WO_3_/TiO_2_ catalyst was also pre-immersed in Mn(NO_3_)_2_ solution for 24 h. Then, the KMnO_4_ solution was gradually added with thorough stirring. The total content of Mn from Mn(NO_3_)_2_ and KMnO_4_ was controlled to be equal to the former two samples. The mixtures were placed at room temperature for 12 h and then filtered followed by washing with de-ionized water for 8–10 times. The filter cake was dried at 50 °C for 3 h and 110 °C for 12 h, respectively. The specific synthesis process of in situ growth method is shown in Figure 1. MnO*_x_* was in-situ grown on the surface of the catalyst, suggested to be from the reaction between KMnO_4_ and Mn(NO_3_)_2_ as the following equation:

(1) $$2KMnO_{4}+3Mn{\left(NO_{3}\right)}_{2}+2H_{2}O\to 5MnO_{2}+4HNO_{3}+2KNO_{3}$$

The obtained catalyst was note as Mn-V-W/Ti-S. For all catalysts prepared by the various methods, the mass fraction of Mn was consistently 9 wt.% in stoichiometric ratio. V_2_O_5_-WO_3_/TiO_2_ is represented by V-W/Ti, the catalysts loaded with Mn are represented by Mn-V-W/Ti, and all samples were crushed and sieved to a 20–40 mesh size (0.425–0.850 mm) for activity measurement.

### 2.3. Catalyst Characterization

The specific surface area of the catalyst was measured using a Quantachrome Nove (2200e) analyzer. The images of the catalysts were characterized using the new Hitachi SU8020 high-resolution field emission scanning electron microscope (SEM). The powder X-ray diffraction (XRD) measurement was carried out on a Rigaku D/MAx2500V system with Cu Kα radiation. Transmission electron microscopy (TEM) images of the samples were obtained by a JEM-2100F (Hitachi, Tokyo, Japan) analyzer. Energy-dispersive X-ray spectroscopy (EDX) was used to determine the element distribution on the surface of catalysts. X-ray photoelectron spectroscopy (XPS) results of the samples were recorded on a scanning X-ray microprobe (ESCACAB250, US Themo Ltd., Waltham, MA, USA). NH_3_-TPD/H_2_-TPR experiments were carried out in the quartz reactor tube and the tubular reaction furnace at atmospheric pressure. The reactor outlet gas during the desorption process was tested on-line by mass spectrometry (Hiden QIC-200). Temperature Programmed Surface Reactions (TPSR) were carried out in the quartz reactor tube and the tubular reaction furnace at atmospheric pressure. Then, 2 g catalyst samples were taken and placed in a quartz tube, passed supersaturated NH_3_ at 100 °C, cut off NH_3_, passed 600 ppm NO, 3% O_2_, Ar as equilibrium gas, and the total gas flow rate is 350 mL·min^−1^, the temperature is raised from 100 °C to 350 °C at a rate of 5 °C·min^−1^, and the gas concentrations of NO were measured by a flue gas analyzer (Testo350-XL).

### 2.4. Catalytic Activity Measurement

The SCR activity tests were conducted in a fixed-bed quartz reactor (i.d. 14 mm). The simulated flue gas consisted of 600 ppm NH_3_, 600 ppm NO, 3 vol.% O_2_, and Ar as the balance. The total flow rate of 350 mL·min^−1^ was maintained for all tests, corresponding to a gas hourly space velocity (GHSV) of 10,500 h^−1^. The gas concentration of NO was measured online by a flue gas analyzer (Testo350-XL). The activity data were collected when the SCR reaction achieved steady state for about 40 min at each temperature. The conversion of NO (X_NO_) is calculated from the following equation:

(2) $$X_{NO}=\frac{{\left[NO\right]}_{in}-{\left[NO\right]}_{out}}{{\left[NO\right]}_{in}^{}}\times 100\%$$

where [NO]_in_ and [NO]_out_ were the inlet and outlet gas concentrations of NO, respectively.

## 3. Results

### 3.1. Catalytic Activity

**Figure 2:**
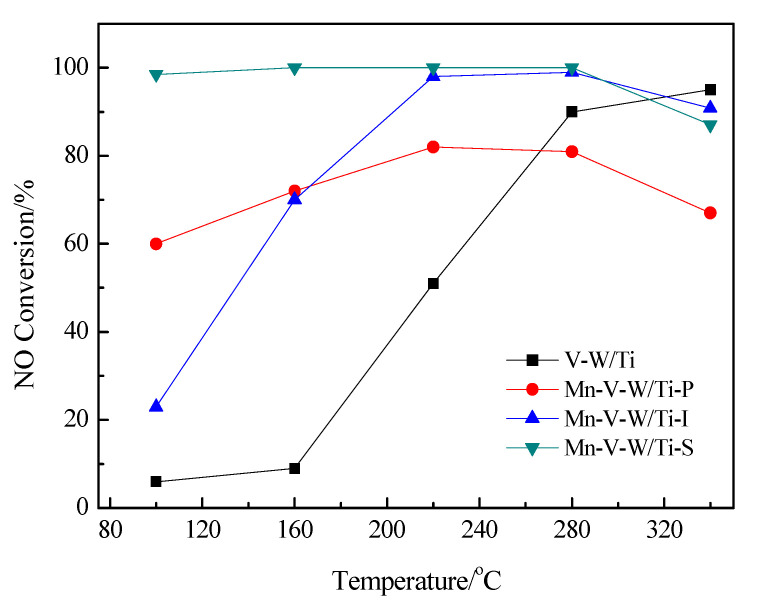
The effect of different preparation methods on catalyst activity. Reaction conditions: [NO] = [NH_3_] = 600 ppm, [O_2_] = 3 vol.%, N_2_ as the balance gas, total flow rate = 350 mL·min^−^^1^.

Figure 2 shows the compared NO conversion on catalysts prepared by three different methods and the original V-W/Ti catalyst. Obviously, the NO conversion of V-W/Ti catalyst without loading Mn exhibits quite lower NO conversion (lower than 50%) at temperatures below 230 °C. After loading Mn by three different methods, the NO conversion of V-W/Ti catalyst at low temperature (100–280 °C) is improved, as shown in Figure 2. Excitingly, the NO conversion of Mn-V-W/Ti-S catalyst obtained by in-situ growth method almost keep 100% in the whole temperature range (100–280 °C). In addition, the activity of Mn-V-W/Ti-S catalyst is higher than that of Mn-V-W/Ti-I catalyst and Mn-V-W/Ti-P catalyst, especially at low temperature below 240 °C. When the temperature is higher than 250 °C, the activity of Mn-V-W/Ti-P catalyst and Mn-V-W/Ti-S catalyst declines. On the contrary, the activity of Mn-V-W/Ti-S keeps. This indicates the Mn-V-W/Ti-S prepared by in-situ growth method is preferred, which not only processes the NO conversion at low temperature contributed by loading Mn but also has the catalytic activity at high temperature contributed by loading V-W.

### 3.2. XRD and SAED Pattern Analysis

**Figure 3:**
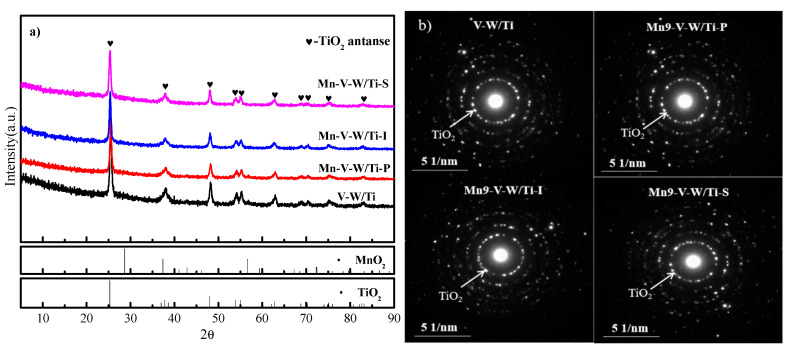
(**a**) XRD pattern of synthesized powder with reference peaks and (**b**) SAED pattern of V-W/Ti and Mn-V-W/Ti.

Figure 3a shows the XRD patterns of the four catalysts. All the catalysts show typical anatase crystal structure (PDF#21-1272) indicating that the catalyst carrier TiO_2_ maintains a relatively good anatase crystal structure and keeps a good stability. After loading Mn, the peak intensity of TiO_2_ weakens a little. There are no differences in the XRD patterns of catalysts prepared by different Mn loading methods. The characteristic peaks of V_2_O_5_, WO_3_, and MnO_x_ were not observed. This may be due to the high dispersion of trace activity components, indicating V_2_O_5_, WO_3,_ and MnO_x_ in the surface of supporter TiO_2_. The dispersion and trace exceed the detected limitation of the measurement. Thus, the characteristic peaks of V_2_O_5_, WO_3_, and MnO_x_ were not observed.

SAED pattern analysis was conducted to confirm whether the MnO_x_ exists in an amorphous state and on the catalyst. Figure 3b shows the diffraction pattern of V-W/Ti and Mn-V-W/Ti. The first diffraction pattern in Figure 3b indicates the TiO_2_ (011) plane, where the measured d-spacing was 3.51 Å. In addition, there is no diffraction pattern of other metal oxides, so the MnO_x_ exists in the form of amorphous forms in the catalyst surface. However, to determine whether the dispersion property of Mn on the catalyst changes depending on the preparation method, we need further research.

### 3.3. TEM and XRF Analysis

**Figure 4:**
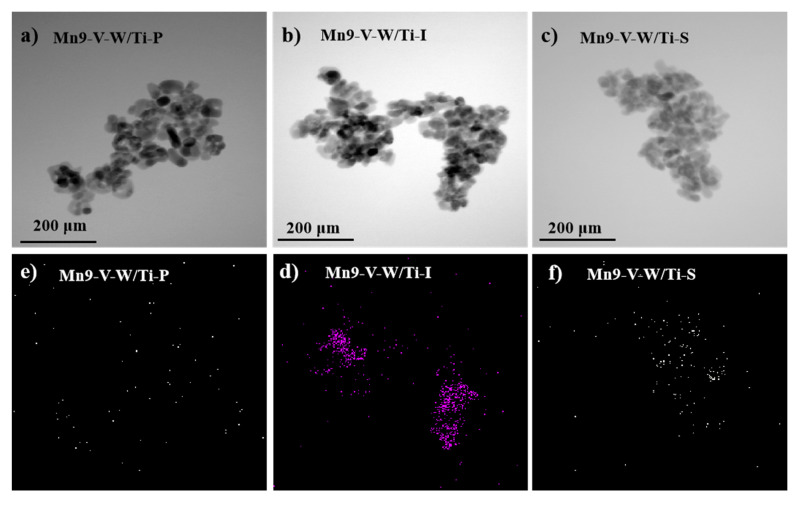
TEM image of the catalyst (**a**–**c**), the dispersion of Mn in the catalyst measured by EDS (**d**–**f**).

In order to clearly observe the morphologies and the dispersion degrees of the active substance on the surface of catalysts, TEM and EDS tests are performed, as shown in Figure 4. It can clearly be seen that the three catalysts have the same microscopic morphology, which is basically characterized by the structural characteristics of titanium dioxide in Figure 4a–c [19].Figure 4d–f show the distribution of Mn on catalysts, and the difference in preparation method has a great influence on the distribution and content of Mn. MnO_x_ had high dispersion in Figure 4f, which means a well-mixed state in all parts of the powder. This shows that Mn can be present on the catalyst in a highly dispersed state by means of in-situ growth. At the same time, we found that the impregnation method has the highest actual manganese loading and the precipitation method is the smallest Figure 4b,d–f and Table 1, which is mainly due to the residual liquid remaining in the latter two preparation methods and containing a certain amount of active component Mn or manganese precursor. Then combined Figure 4 with Table 2, it’s also interesting to find that high dispersibility can effectively increase the specific surface area of the catalyst and pore volume. On the contrary, it can be seen from the EDS spectrum in Figure 4d that the MnO*_x_* on the surface of the Mn-V-W/Ti-I catalyst has obvious agglomeration, which leads to a significant decrease in the specific surface area (Table 2). It is seen from Figure 4d that the Mn on the surface of the Mn-V-W/Ti-I catalyst is mostly piled up and is the worst of the three catalysts; therefore, the superior NO conversion of the catalyst has an important relationship with the dispersion of the active component. Impregnation is a one-step impregnation which, in itself, will lead to uneven loading during calcination. As a result, although the impregnation method has the highest actual loading, low dispersion of Mn on catalyst surface will also decrease its NO conversion.

### 3.4. SEM and BET Analysis

**Figure 5:**
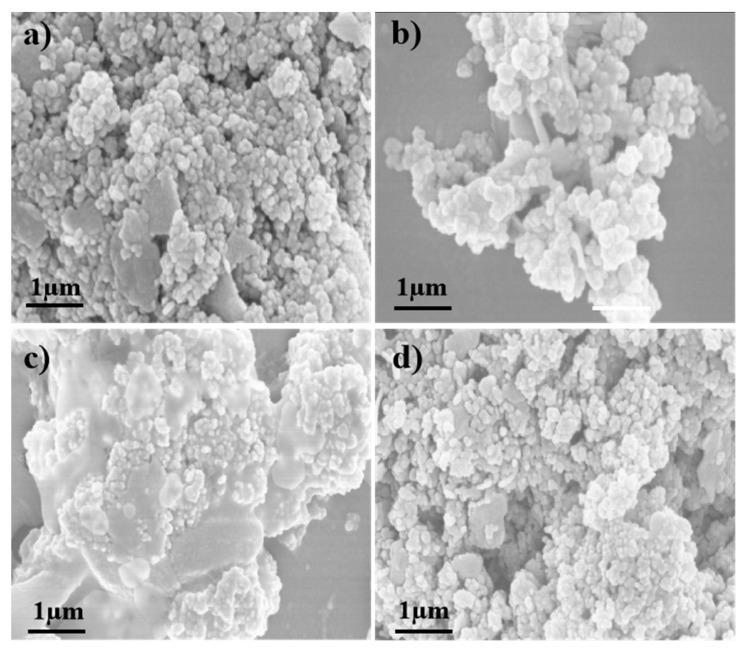
SEM images of the catalysts: (**a**)V-W/Ti; (**b**)Mn-V-W/Ti-P; (**c**)Mn-V-W/Ti-I; (**d**)Mn-V-W/Ti-S.

The SEM images of the four catalysts are shown in Figure 5. It can be seen that the surface morphology of the catalyst V-W/Ti (Figure 5a) is spherical and the particles are evenly distributed. The image of the catalyst keeps a spherical structure after doping metal Mn, indicating that the carrier structure is not destroyed by the addition of MnO_x_. This is consistent with the results of XRD. However, the catalyst particles tend to agglomerate and the agglomeration degree of catalysts prepared by different methods is quite different. The agglomeration of Mn-V-W/Ti-I (Figure 5b) and Mn-V-W/Ti-P (Figure 5c) catalysts is the most serious, which is due to the inevitable sintering of the catalyst surface during high-temperature calcination. While the agglomeration of catalyst Mn-V-W/Ti-S (Figure 5d) is better significantly with better dispersion, this is mainly due to the in-situ reaction at room temperature.

Table 2 shows the BET data for the four catalysts. As shown in Table 2, the specific surface area and pore volume of catalysts obviously changed after doping Mn. The order of specific surface area is Mn-V-W/Ti-S > Mn-V-W/Ti-P > Mn-V-W/Ti-I, and the order of pore volume is Mn-V-W/Ti-S > Mn-V-W/Ti-I > Mn-V-W/Ti-P. Compared with the three preparation methods, the catalyst prepared by in-situ growth has the largest specific surface area (91.3 m^2^/g) and pore volume (0.266 cm^3^/g). This may be caused by the distribution of Mn in catalysts obtained by different preparation method. As for Mn-V-W/Ti-S catalyst, the distribution of Mn in this catalyst is best as shown in SEM and TEM results, leading to a high BET surface area and pore volume. Notably, the high BET surface area and pore volume of the catalyst are beneficial for catalytic conversion of NO. As a result, more activity sites are provided for the catalytic process [20].The BET results in Table 2 are well in accordance with the NO conversion in Figure 2. It is also worth noting that three catalysts of average pore size increased gradually in the order of Mn-V-W/Ti-S, Mn-V-W/Ti-P, and Mn-V-W/Ti-I. This may be due to high-temperature calcination of precipitation and the impregnation method will cause the pore wall to collapse; therefore, some small mesopores become larger mesopores and the original large mesopores directly reach the large pores, even though its average pore size is relatively small.

### 3.5. XPS Analysis

**Figure 6:**
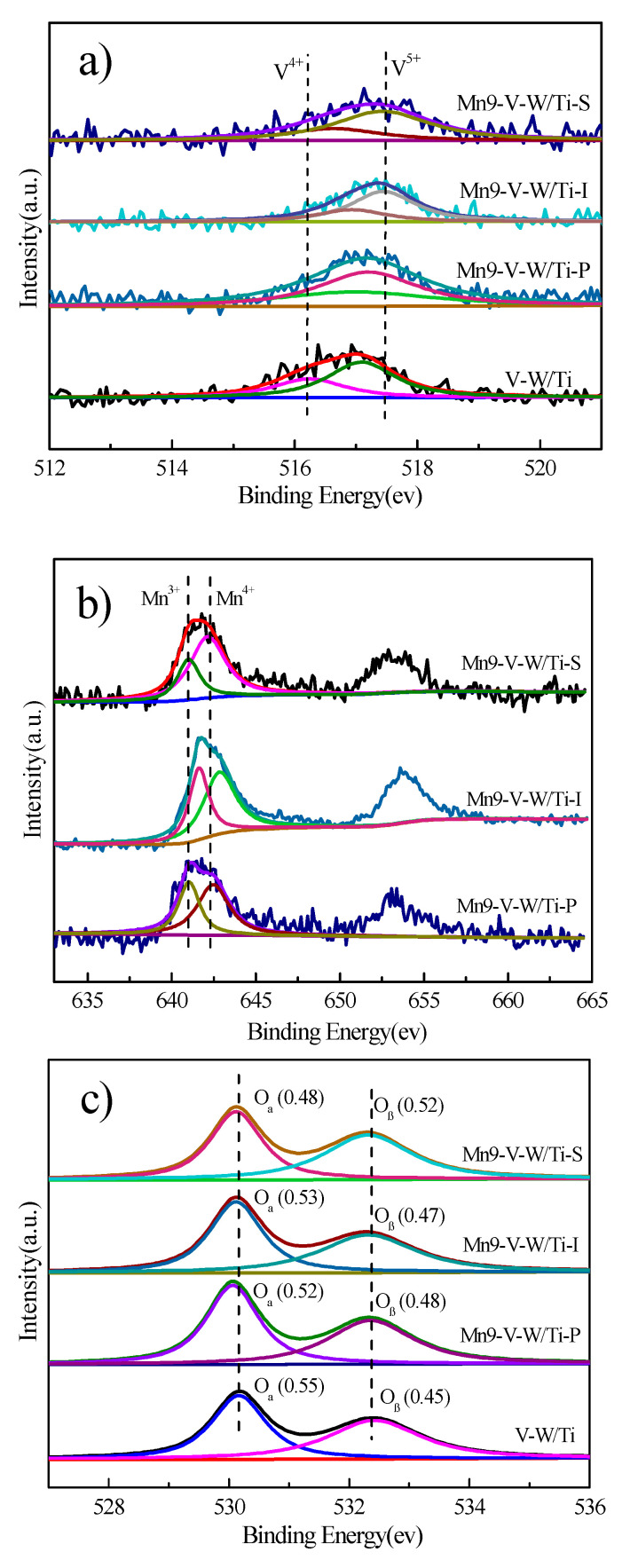
(**a**) V2p; (**b**) Mn2p; (**c**) O1s XPS spectra of the catalysts.

The concentration of surface elements and their chemical states in the SCR catalyst plays an important role in the SCR reaction and can be determined by XPS analysis. The XPS analysis results are shown in Figure 6.

Figure 6a shows the V2p_3/2_ orbital spectrum of Mn-V-W/Ti and V-W/Ti catalysts, which can be fitted to V^4+^ and V^5+^ peaks. Specific peak positions and chemical concentrations of V elements shown in Table 3. Among them, V^5+^ in the V-W/Ti catalyst accounts for 0.7 of the V element, while the V^5+^ content in the Mn-V-W/Ti-S, Mn-V-W/Ti-P, and Mn-V-W/Ti-I catalysts are 0.79, 0.75, and 0.78, respectively. Moreover, it is also apparent from Figure 4 that the V2p_3/2_ orbital peak position of the Mn-V-W/Ti catalyst is significantly shifted toward the direction of high binding energy with respect to the V-W/Ti catalyst, which may be due to the doping of Mn and the average valence is rising, and this conclusion is consistent with the change in V^5+^ content.

Figure 6b presents the Mn 2p XPS spectra for Mn-V-W/Ti. The two main peaks located at about 642 eV and 653 eV could be ascribed to Mn 2p_3/2_ and Mn 2p_1/2_, respectively [21,22]. The peak fitting of the Mn2p_3/2_ orbital can be fitted to the Mn^4+^ peak and the Mn^3+^ peak and specific peak position and the chemical concentration of the Mn element are shown in Table 3. Since Mn^4+^ has a higher valence in manganese oxide, it can promote the conversion of NO to NO_2_, which promotes the rapid SCR reaction [23]. Therefore, Mn^4+^ has an important influence on the catalytic performance of the catalyst. Comparing Mn-V-W/Ti-S, Mn-V-W/Ti-P, and Mn-V-W/Ti-I catalysts, it can be found that the difference in the preparation method makes the chemical valence of Mn elements more obvious, among which the Mn^4+^ content in the Mn-V-W/Ti-S catalyst is the highest. Many researchers have well demonstrated that the high oxidation state of manganese (Mn^4+^) is one of the key factors to promote NO conversion of the catalyst [24], so the catalyst prepared by the in-situ growth method has the highest Mn^4+^ content (0.7) among the three preparation method, which makes it have better low-temperature NO removal rate of ability. This is closely related to the strong redox reaction of KMnO_4_ and Mn(NO_3_)_2_ in the in-situ growth method converts more Mn^2+^ into Mn^4+^, while the other two methods are relatively less.

Additionally, O1s spectra for V-W/Ti and Mn-V-W/Ti catalysts are exhibited in Figure 6c. O1s peaks can be separated into two groups: lattice oxygen (O_α_) and surface adsorption oxygen (O_β_), specific peak positions and chemical concentrations of O elements shown in Table 3 [25,26]. It can be seen from the table that the proportion of O_β_ oxygen in the Mn-V-W/Ti-S catalyst is as high as 50%. We know that Mn^4+^ can promote the oxidation of NO to NO_2_, and the adsorption of oxygen on the catalyst surface can accelerate the oxidation efficiency of NO due to its large fluidity, thereby facilitating the progression of a rapid SCR response.

### 3.6. H_2_-TPR Analysis

**Figure 7:**
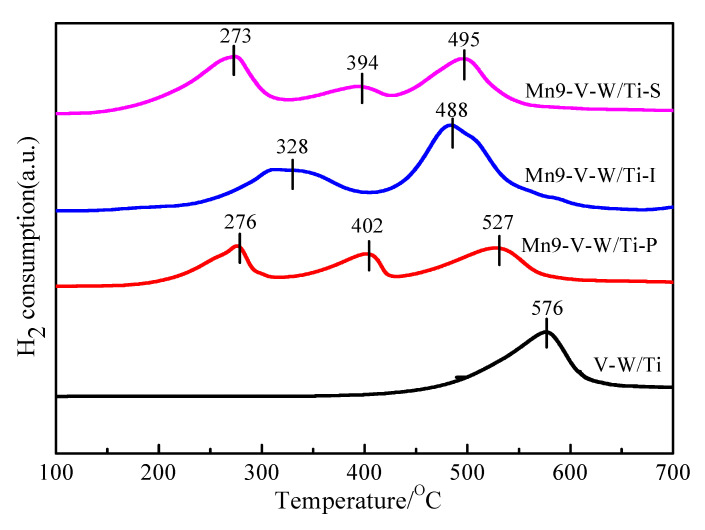
H_2_-temperature programmed reduction (TPR) profiles of the catalysts. Reaction conditions: purging the catalysts with N_2_ at 110 °C; programmed temperature reduction with H_2_ at 50–600 °C, the heating rate is 5 °C/min.

The redox properties of a catalyst play an important role in the low-temperature NH_3_-SCR performance over metal oxides [27,28]. The H_2_-TPR profiles recorded over the V-W/Ti, Mn-V-W/Ti-S, Mn-V-W/Ti-I, and Mn-V-W/Ti-P catalysts are shown in Figure 7. The V-W/Ti catalyst showed a distinct characteristic peak at 576 °C, which is attributed to the reduction peak of V^5+^→V^4+^ [29]. For the modified catalysts Mn-V-W/Ti-S and Mn-V-W/Ti-P, there are three reduction peaks: the former two peaks are the continuous reduction steps of Mn^4+^→Mn^3+^→Mn^2+^ [30], and the third one is the V^5+^→V^4+^ characteristic reduction peak. As for the profiles of Mn-V-W/Ti-I catalyst, there are only two reduction peaks here: the former is related to the co-reduction step of Mn^4+^→Mn^3+^ and Mn^3+^→Mn^2+^, and the latter is the V^5+^→V^4+^ characteristic reduction peak. Here we can clearly see that no matter how the Mn is introduced into the V-W/Ti catalyst, the reduction peak of V^5+^ moves toward the low temperature (576 °C→488 °C, 495 °C, 527 °C). The interaction between V and Mn species enhances the reduction of vanadium, resulting in better redox properties of Mn-V-W/Ti. In addition, it was observed that the lowest Mn^4+^ reduction peak temperature appears in Mn-V-W/Ti-S, indicating that the oxidation capacity is the highest. This may be due to the fact that the catalyst prepared by the low-temperature in-situ growth method does not cause sintering, and MnO_x_ is dispersed in the catalyst carrier evenly. The surface dispersibility is good, so that the active component is more easily contacted with the reducing gas H_2_.

### 3.7. NH_3_-TPD Analysis

**Figure 8:**
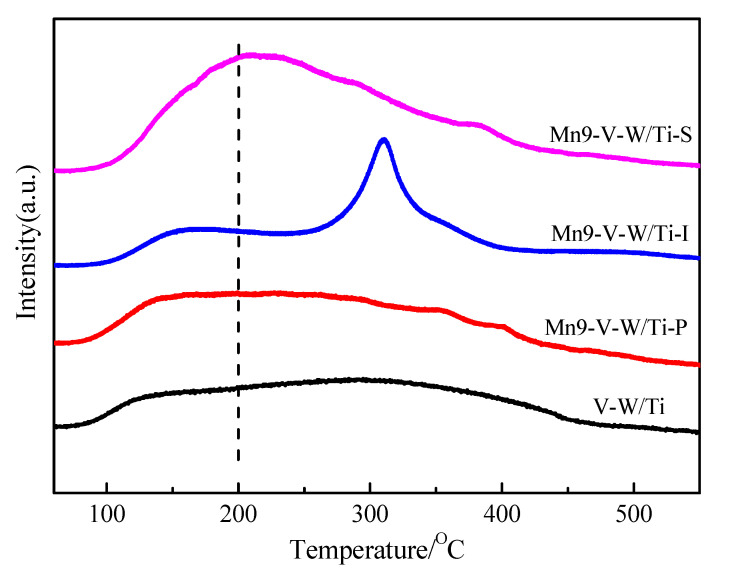
NH_3_-TPD profiles of the catalysts. Reaction conditions: purging the catalysts with N_2_ at 110 °C; NH_3_ was adsorbed at 50 °C; NH_3_ was desorption at 50–550 °C, the heating rate is 5 °C/min.

NH_3_ adsorption is critical for SCR reactions, which is related to the surface acidity of the SCR catalyst [31]. Figure 8 shows the NH_3_-TPD curve of the four catalysts. The low-temperature desorption peak below 200 °C is attributed to the weak acidic site, and the broad peak between 200 °C and 450 °C belongs to the strong acid site [32].

Below 200 °C, the Mn-V-W/Ti catalyst is transferred to the higher temperature range than the V-W/Ti catalyst, this indicates that the surface of the catalyst has relatively strong acidity after loading Mn. According to the SCR reaction mechanism, the strong acid sites on the surface of the catalyst are beneficial to the NH_3_ adsorption and activation at low temperature, and improve the low-temperature catalytic activity [33]. Among them, the desorption peak of the catalyst prepared by the in-situ growth method has a large degree of deviation, which appears at about 220 °C (belonging to a strong acidic site). Moreover, the desorption peak area of Mn-V-W/Ti-S is the largest among the catalysts prepared by the three methods. After the temperature exceeds 250 °C, the desorption peaks of Mn-V-W/Ti-S and Mn-V-W/Ti-P catalysts become weaker, while the V-W/Ti and Mn-V-W/Ti-I catalysts have a larger desorption peak about 300 °C, which indicates that the reason for the decrease of the activity of the in-situ growth method at high temperature is probably caused by the large decrease in the adsorption capacity of NH_3_ at high temperature. For the impregnation method, it has high activity at high temperature, which is related to the high NH_3_ adsorption of Mn-V-W/Ti-I catalyst at high temperature.

### 3.8. TPSR Analysis

In order to more intuitively explore the performance of the four catalysts in the SCR reaction after pre-adsorption of NH_3_, temperature programmed surface reaction (TPSR) was used for the study. Each catalyst sample was treated with 600 ppm NH_3_ for 1 h, then treated with N_2_ for 30 min, and finally 600 ppm of NO and 3% of O_2_ were introduced into the catalyst to carry out the reaction.

**Figure 9:**
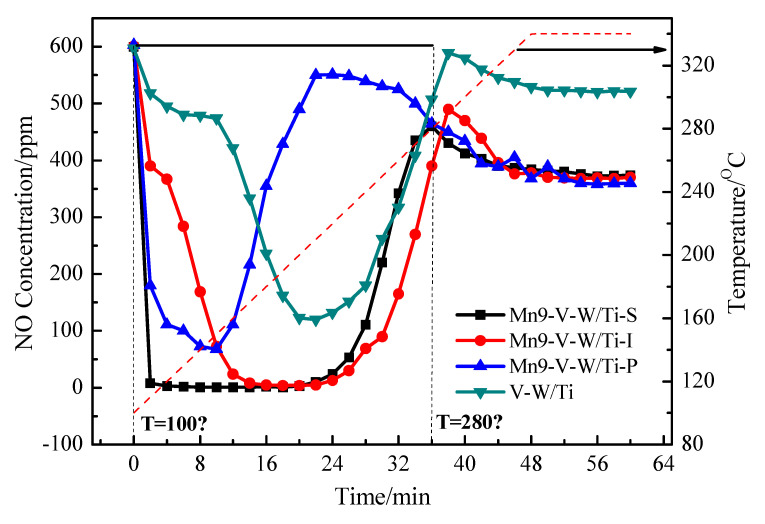
Temperature programmed surface reaction (TPSR) profiles of the catalysts at a space velocity of 10,500 h^−^^1^. Reaction conditions: [NO] = [NH_3_] = 600 ppm, [O_2_] = 3 vol.%, N_2_ as the balance gas, total flow rate = 350 mL·min^−^^1^.

The result is shown in Figure 9, the four catalysts showed a significant inverted peak on the TPSR curve after pre-adsorption by NH_3_, which was caused by the surface reaction of NO with the adsorbed NH_3_. When the temperature of the system is finally stabilized at 340 °C, the NO outlet concentration of all the catalysts is gradually reduced to a concentration lower than the inlet, which is caused by the heat consumption of NO. It is obvious that the NO outlet concentration of the V-W/Ti catalyst decreases slowly in 0–10 min and the NO concentration decreases rapidly after 10 min. Therefore, the NH_3_ adsorbed by the V-W/Ti catalyst is sufficient to react with NO within 0–10 min, but in fact the NH_3_ participating in the reaction is less, so the V-W/Ti catalyst has a weak SCR reaction at low temperature. The NO outlet concentration of the Mn-V-W/Ti catalyst decreases rapidly in the same time. The degree of surface reaction of the catalyst caused by the different preparation methods is different, for the Mn-V-W/Ti-S catalyst, the NO outlet concentration is close to 0 ppm after 2 min of reaction, which is the most efficient of the three methods. Comparing the V-W/Ti and Mn-V-W/Ti catalysts, we can see that the introduction of Mn^n+^ enhances the low-temperature SCR reaction ability of the catalyst. This ability mainly comes from the oxidation ability of the transition metal Mn. Combined with XPS, the higher the amount of valence Mn, the stronger the reactivity. The catalyst prepared by the in-situ growth method has more Mn^4+^, so the catalyst Mn-V-W/Ti-S has a faster and more efficient ability to remove NO at a low temperature, and the result is consistent with the results of the activity test. At the same time, we integrated the inverted peaks of the four catalysts on the curve and related calculations (0–36 min) and obtained that the treatment capacity of NO for Mn-V-W/Ti-S, Mn-V-W/Ti-I, Mn-V-W/Ti-P, and V-W/Ti catalysts after pre-adsorption of ammonia was 1.5 × 10^−^^4^ mol/g, 1.23 × 10^−^^4^ mol/g, 0.68 × 10^−^^4^ mol/g, 0.67 × 10^−^^4^ mol/g. Therefore, the catalyst prepared by the in-situ growth method has a faster and more efficient ability to remove NO_x_ at a low temperature, and the results are consistent with the results of the activity test.

## 4. Conclusions

Compared with the traditional impregnation method and the precipitation method, the modified catalyst prepared by the in-situ growth method has superior low-temperature SCR performance. The conversion rate of NO reached 98% at 100 °C, and the conversion rate of 100% was always maintained from 100–280 °C, with a wide window of high activity temperature. The results of XRF show that the Mn loading efficiency of Mn-V-W/Ti-S catalyst is lower than that of Mn-V-W/Ti-I catalyst, but its NO conversion is much higher than that of Mn-V-W/Ti-I catalyst at low temperature. The main reason is divided into two aspects: On the one hand, the preparation conditions of Mn-V-W/Ti-S catalyst are normal temperature, the surface of the catalyst does not appear to be sintered, and has a large specific surface area, which provides a basis for the high dispersibility of MnO_x_ on the catalyst surface. It can also concluded from H_2_-TPR that it is because of this high dispersibility that the active component in the Mn-V-W/Ti-S catalyst is more easily contacted with the gas phase to make the catalyst have higher oxidation capacity. On the other hand, the results of NH_3_-TPD and XPS show that the Mn-V-W/Ti-S catalyst has more acidic sites, Mn^4+^, and surface adsorbed oxygen which are favorable for NH_3_ adsorption; therefore, the Mn-V-W/Ti-S catalyst can maintain a 100% NO removal rate within 0–20 min in the TPSR.

## Figures and Tables

**Table 1:** X Ray Fluorescence of the catalyst.

Sample	Mn(%)
Mn-V-W/Ti-P	4.42
Mn-V-W/Ti-I	7.09
Mn-V-W/Ti-S	6.45

**Table 2:** The specific surface area, pore volume, and average pore size of the catalysts.

Sample	S_BET_ (m^2^/g)	Pore Volume (cm^3^/g)	Average Pore Size (nm)
V-W/Ti	90.5	0.441	15.6
Mn-V-W/Ti-P	58.9	0.153	11.4
Mn-V-W/Ti-I	38.9	0.218	11.2
Mn-V-W/Ti-S	91.3	0.266	11.1

**Table 3:** The V2p and Mn2p XPS results of various catalysts.

Sample	Binding Energy(ev)				Mn^4+^/Mn	V^5+^/V
	Mn^4+^	Mn^3+^	V^5+^	V^4+^		
V-W/Ti	/		517.09	516.24	/	0.70
Mn-V-W/Ti-P	642.47	641.03	517.19	516.97	0.59	0.75
Mn-V-W/Ti-I	642.83	641.61	517.44	516.93	0.58	0.78
Mn-V-W/Ti-S	642.13	641.00	517.12	516.32	0.70	0.79

## References

[1] Xu W.J., Zhang G.X., Chen H.W., Zhang G.M., Han Y., Chang Y.C., Gong P. (2018). Mn/beta and Mn/ZSM-5 for the low-temperature selective catalytic reduction of NO with ammonia: Effect of manganese precursors. Chin. J. Catal..

[2] Park E., Chin S., Jeong J., Jurng J. (2012). Low-temperature NO oxidation over Mn/TiO_2_, nanocomposite synthesized by chemical vapor condensation: Effects of Mn precursor on the surface Mn species. Microporous Mesoporous Mater..

[3] Ji P.D., Gao X., Du X.S., Zheng C.H., Luo Z.Y., Cen K.F. (2016). Relationship between the molecular structure of V_2_O_5_/TiO_2_ catalysts and the reactivity of SO_2_ oxidation. Catal. Sci. Technol..

[4] Zhao X., Hang L., Namuangruk S., Hu H., Hu X.N., Shi L.Y., Zhang D.S. (2016). Morphology dependent performance of Zr-CeVO_4_/TiO_2_ for selective catalytic reduction of NO with NH_3_. Catal. Sci. Technol..

[5] Ding S.P., Liu F.D., Shi X.Y., Liu K., Lian Z.H., Xie L.J., He H. (2015). Significant Promotion Effect of Mo Additive on a Novel Ce-Zr Mixed Oxide Catalyst for the Selective Catalytic Reduction of NO*_x_* with NH_3_. ACS Appl. Mater. Interfaces.

[6] Li B., Ren Z.Y., Ma Z.X., Huang X.D., Liu F., Zhang X.B., Yang H.S. (2016). Selective catalytic reduction of NO by NH_3_ over CuO-CeO_2_ in the presence of SO_2_. Catal. Sci. Technol..

[7] Huang Z.G., Zhu Z.P., Liu Z.Y. (2002). Combined effect of H_2_O and SO_2_ on V_2_O_5_/AC catalysts for NO reduction with ammonia at lower temperatures. Appl. Catal. B Environ..

[8] Qi K., Xie J.L., Fang D., Li F.X., He F. (2017). Performance enhancement mechanism of Mn-based catalysts prepared under N_2_ for NO*_x_* removal: Evidence of the poor crystallization and oxidation of MnO*_x_*. Chin. J. Catal..

[9] Zuo J.L., Chen Z.G., Wang F.R., Yu Y.H., Wang L.F., Li X.H. (2014). Low-Temperature Selective Catalytic Reduction of NO*_x_* with NH_3_ over Novel Mn–Zr Mixed Oxide Catalysts. Ind. Eng. Chem. Res..

[10] Wallin M., Forser S., Thormählen P., Skoglundh M. (2004). Screening of TiO_2_-supported catalysts for selective NO*_x_* reduction with ammonia. Ind. Eng. Chem. Res..

[11] Qi G., Yang R.T. (2003). Performance and kinetics study for low-temperature SCR of NO with NH_3_ over MnO*_x_*-CeO_2_ catalyst. J. Catal..

[12] Liu Z.M., Li Y., Zhu T.L., Su H., Zhu J.Z. (2014). Selective catalytic reduction of NO*_x_* by NH_3_ over Mn-Promoted V_2_O_5_/TiO_2_ Catalyst. Ind. Eng. Chem. Res..

[13] Zhao X., Huang L., Li H.L., Gu H., Han J., Shi L.Y. (2015). Highly dispersed V_2_O_5_/TiO_2_, modified with transition metals (Cu, Fe, Mn, Co) as efficient catalysts for the selective reduction of NO with NH_3_. Chin. J. Catal..

[14] Wu Z.B., Jiang B.Q., Liu Y. (2008). Effect of transition metals addition on the catalyst of manganese/titania for low-temperature selective catalytic reduction of nitric oxide with ammonia. Appl. Catal. B Environ..

[15] Li J.H., Chen J.J., Ke R., Luo C.K., Hao J.M. (2007). Effects of precursors on the surface Mn species and the activities for NO reduction over MnO*_x_*/TiO_2_ catalysts. Catal. Commun..

[16] Zuo H.Q., Xu D.Y., Liu W., Dan H.J., Liu X.H., Lin S., Hou P. (2018). Heat-treated Dolomite-palygorskite clay supported MnO*_x_* catalysts prepared by various methods for low temperature selective catalytic reduction (SCR) with NH_3_. Appl. Clay Sci..

[17] Qi G., Yang R.T. (2003). Low-temperature selective catalytic reduction of NO with NH_3_ over iron and manganese oxides supported on titania. Appl. Catal B Environ..

[18] Zhang H.G., Li X.Z., Su H., Chen X.F., Zuo S.X., Yan X.Y., Liu W.J., Yao C. (2019). Sol–gel synthesis of upconversion perovskite/attapulgite heterostructures for photocatalytic fixation of nitrogen. J. Sol-Gel Sci. Technol..

[19] Jo S., Shin B., Shin M.-C., Tyne C., Lee H. (2014). Dispersion and valence state of MnO_2_/Ce*_(1-x)_*Zr*_x_*O_2_–TiO_2_ for low temperature NH_3_-SCR. Catal. Commun..

[20] Xiong Z.B., Lu C.M. (2013). Study on the modification of iron-cerium mixed oxide catalyst for selective catalytic reduction of NO. J. Fuel Chem. Technol..

[21] Kang M., Park E.D., Kim J.M., Yie J.E. (2007). Manganese oxide catalysts for NO*_x_* reduction with NH_3_, at low temperatures. Appl. Catal. A Gen..

[22] Tian W., Yang H.S., Fan X.Y., Zhang X.B. (2011). Catalytic reduction of NO*_x_* with NH_3_ over different-shaped MnO_2_ at low temperature. J. Hazard. Mater..

[23] Zhu S., Shen B., Chi G., Zhang X. (2017). Low-temperature SCR of NO over Fe and Co co-doped Mn-Ce/TiO_2_ catalyst. Chin. J. Environ. Eng..

[24] Chao M.X., Mao D.S., Li G.H., Li G., Yu J., Guo X.M. (2020). Low-temperature selective catalytic reduction of NO with NH_3_ over Mn–Ce–Ox/TiO_2_: A comparison between catalyst preparation methods. J. Sol-Gel Sci. Technol..

[25] Fang D., He F., Xie J.L. (2019). Characterization and performance of common alkali metals and alkaline earth metals loaded Mn/TiO_2_ catalysts for NO*_x_* removal with NH_3_. J. Energy Inst..

[26] Shu Y., Sun H., Quan X., Chen S. (2012). Enhancement of catalytic activity over the iron-modified Ce/TiO_2_ catalyst for selective catalytic reduction of NO*_x_* with ammonia. J. Phys. Chem. C.

[27] Lietti L. (1996). Reactivity of V_2_O_5_–WO_3_/TiO_2_ de-NO*_x_* catalysts by transient methods. Appl. Catal. B. Environ..

[28] Ramis G., Yi L., Busca G., Turco M., Kotur E., Willey R.J. (1995). Adsorption, activation, and oxidation of ammonia over SCR catalysts. J. Catal..

[29] Putluru S.S.R., Riisager A., Fehrmann R. (2009). The Effect of acidic and redox properties of V_2_O_5_/CeO_2_-ZrO_2_ catalysts in selective catalytic reduction of NO by NH_3_. Catal. Lett..

[30] Zhang Q.L., Qiu C.T., Xu H.D., Lin T., Lin Z.E., Gong M.C., Chen Y.Q. (2011). Low-temperature selective catalytic reduction of NO with NH_3_ over monolith catalyst of MnO*_x_*/CeO_2_–ZrO_2_–Al_2_O_3_. Catal. Today.

[31] Guo R.T., Wang Q.S., Pan W.G., Chen Q.L., Ding H.L., Yin X.F., Yang N.Z., Lu C.Z., Wang S.X., Yuan Y.C. (2015). The poisoning effect of heavy metals doping on Mn/TiO_2_ catalyst for selective catalytic reduction of NO with NH_3_. J. Mol. Catal. A Chem..

[32] Zhang Q.M., Song C.L., Lv G., Bin F., Pang H.T., Song J.O. (2015). Effect of metal oxide partial substitution of V_2_O_5_, in V_2_O_5_–WO_3_/TiO_2_, on selective catalytic reduction of NO with NH_3_. Ind. Eng. Chem..

[33] Shen M.Q., Li C.X., Wang J.Q., Xu L.L., Wang W.L., Wang J. (2015). New insight into the promotion effect of Cu doped V_2_O_5_/WO_3_–TiO_2_ for low temperature NH_3_-SCR performance. RSC Adv..

